# In vivo evaluation of a hyperspectral imaging system for minimally invasive surgery (HSI-MIS)

**DOI:** 10.1007/s00464-023-09874-2

**Published:** 2023-01-16

**Authors:** Madeleine T. Thomaßen, Hannes Köhler, Annekatrin Pfahl, Sigmar Stelzner, Matthias Mehdorn, René Thieme, Boris Jansen-Winkeln, Ines Gockel, Claire Chalopin, Yusef Moulla

**Affiliations:** 1 Department of Visceral, Transplant, Thoracic and Vascular Surgery, University Hospital of Leipzig, Liebigstr. 20, 04103 Leipzig, Germany; 2 Innovation Center Computer Assisted Surgery (ICCAS), Faculty of Medicine, Leipzig University, Semmelweisstr. 14, 04103 Leipzig, Germany; 3 Department of General, Visceral, Thoracic, and Vascular Surgery, Klinikum St. Georg, 04129 Leipzig, Germany

**Keywords:** Hyperspectral imaging, Minimally invasive surgery, Clinical evaluation study, Gastrointestinal surgery, Laparoscopic surgery, Perfusion

## Abstract

**Background:**

Hyperspectral Imaging (HSI) is a reliable and safe imaging method for taking intraoperative perfusion measurements. This is the first study translating intraoperative HSI to an in vivo laparoscopic setting using a CE-certified HSI-system for minimally invasive surgery (HSI-MIS). We aim to compare it to an established HSI-system for open surgery (HSI-Open).

**Methods:**

Intraoperative HSI was done using the HSI-MIS and HSI-Open at the Region of Interest (ROI). 19 patients undergoing gastrointestinal resections were analyzed in this study. The HSI-MIS-acquired images were aligned with those from the HSI-Open, and spectra and parameter images were compared pixel-wise. We calculated the Mean Absolute Error (MAE) for Tissue Oxygen Saturation (StO_2_), Near-Infrared Perfusion Index (NIR-PI), Tissue Water Index (TWI), and Organ Hemoglobin Index (OHI), as well as the Root Mean Squared Error (RMSE) over the whole spectrum. Our analysis of parameters was optimized using partial least squares (PLS) regression. Two experienced surgeons carried out an additional color-change analysis, comparing the ROI images and deciding whether they provided the same (acceptable) or different visual information (rejected).

**Results:**

HSI and subsequent image registration was possible in 19 patients. MAE results for the original calculation were StO_2 orig._ 17.2% (± 7.7%)_,_ NIR-PI_orig._ 16.0 (± 9.5), TWI_orig._ 18.1 (± 7.9), OHI_orig._ 14.4 (± 4.5). For the PLS calculation, they were StO_2 PLS_ 12.6% (± 5.2%), NIR-PI_PLS_ 10.3 (± 6.0), TWI_PLS_ 10.6 (± 5.1), and OHI_PLS_ 11.6 (± 3.0). The RMSE between both systems was 0.14 (± 0.06). In the color-change analysis; both surgeons accepted more images generated using the PLS method.

**Conclusion:**

Intraoperative HSI-MIS is a new technology and holds great potential for future applications in surgery. Parameter deviations are attributable to technical differences and can be reduced by applying improved calculation methods. This study is an important step toward the clinical implementation of HSI for minimally invasive surgery.

Anastomotic leakage (AL) remains a feared complication in gastrointestinal surgery, potentially inducing peritonitis, severe sepsis, followed by a complex deterioration in patients’ recovery. It can lead to higher morbidity, mortality, and a higher risk for permanent stoma formation when preceded by colorectal surgery [1–7]. An adequate blood supply to the anastomosis is a significant success factor in preventing AL. Surgeons usually rely on subjective evaluation of factors like bleeding from marginal vessels, pulsation of arteries, and tissue color to evaluate perfusion. Intraoperative perfusion-testing via imaging methods can reduce the occurrence of AL and improve outcomes [4]. Recent research demonstrates the applicability of IndoCyanine Green-Fluorescence Angiography (ICG-FA) for this purpose [8–12]. It can, however, trigger allergies and anaphylaxis, and is unsuitable for patients with thyroid gland diseases, renal failure, and in pregnant women.

Hyperspectral Imaging (HSI) enables spectroscopy for every image pixel. Metabolites reflect light at distinct wavelengths, e.g., oxygenated and deoxygenated hemoglobin differ in their light-absorption characteristics. The data collected can be used to take non-invasive, spatially resolved perfusion measurements [11, 13]. Previous studies reported safe and reliable results after using the HSI-system for open surgery (HSI-Open) TIVITA® Tissue (Diaspective Vision GmbH, Am Salzhaff-Pepelow, Germany), especially when determining the resection margin in colorectal surgery, or the ideal anastomotic position of the gastric conduit when constructing esophagogastric anastomoses [14–16]. It has also been successfully evaluated for detecting intestinal ischemia [13, 17, 18], wound monitoring [19–25], graft assessment in transplant surgery [26], automated cancer diagnosis [27–34], and cancer surgical margin delineation [35], as well as for the identification of anatomic structures [36–40]. So far, its use has been limited to open surgery because of the size of current cameras.

In daily routine, most gastrointestinal anastomoses are done laparoscopically or robotically. Previous limitations of intraoperative HSI application, such as large camera size and long data acquisition times, have been overcome by the CE-certified HSI-system for minimally invasive surgery (HSI-MIS) TIVITA® Mini Endoscopy Edition (Diaspective Vision GmbH, Am Salzhaff-Pepelow, Germany) [41]. But before it can be implemented in routine surgery, it needs to be evaluated in comparison with the conventional HSI-Open to determine any differences between the systems and to see whether the HSI-Open’s findings can be translated to the laparoscopic system.

The objective of this study was to compare the HSI-MIS with an established method. We aimed to detect differences between the systems and determine whether the new system is suitable for perfusion measurements in a minimally invasive setting.

## Methods

### The principle of hyperspectral imaging (HSI)

**Fig. 1:**
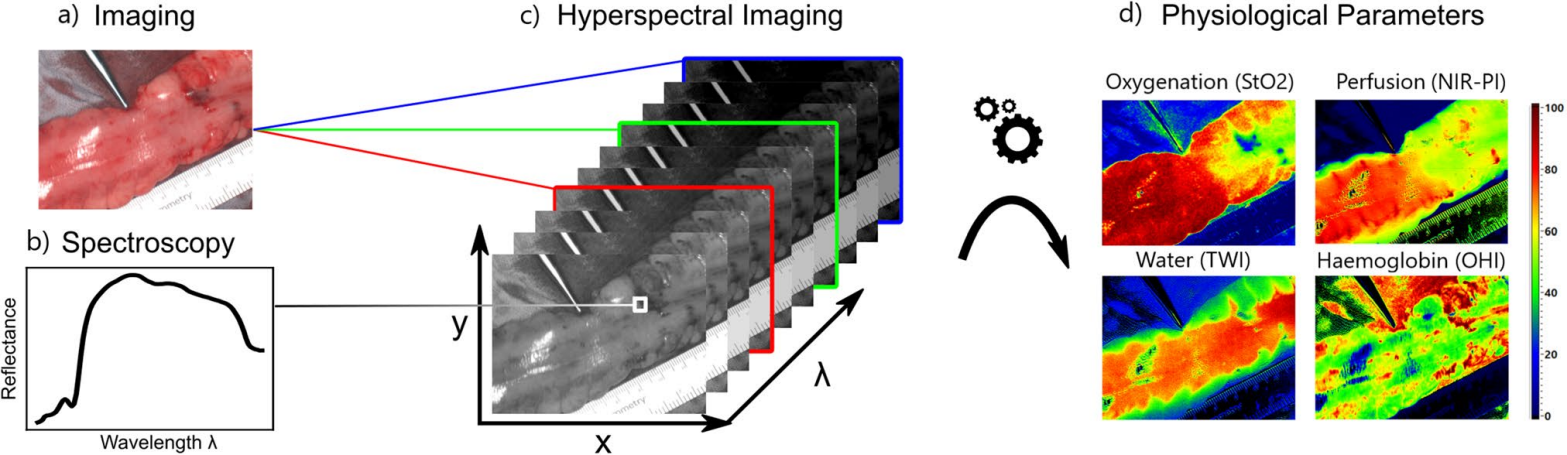
The Region of Interest (ROI) is imaged, and a reconstructed color image is provided (**a**). Spectroscopy (**b**) is performed for every single pixel, creating the so-called hypercube (**c**). The raw spectral data are used for computing the parameters (**d**) for Oxygenation (StO_2_), Near InfraRed-Perfusion Index (NIR-PI), Tissue Water Index (TWI), and Organ Hemoglobin Index (OHI)

Hyperspectral images are defined as images where a broadband spectrum of electromagnetic waves is acquired for every single pixel. The information is visualized as a so-called hypercube with two spatial (*x* and *y*) and one spectral dimension (*λ*) (Fig. 1) [42]. The interaction of metabolites and molecules with light is distinct at different wavelengths, creating a unique pattern. Each pixel’s spectral information reflects the substances present in that area. Each organ and tissue, therefore, creates its distinct spectral fingerprint. Computing power and advanced image-processing techniques have evolved rapidly over the last few years [43], enabling the interpretation of this immense spectral information by translating it into tissue parameters that are easy to understand. For surgical purposes, the manufacturer provides for example Oxygen Saturation (StO_2_), and the Near InfraRed-Perfusion Index (NIR-PI), which enables perfusion measurements at a deeper tissue level, the Tissue Water Index (TWI) and Organ Hemoglobin Index (OHI), a proxy for erythrocyte numbers regardless of the given tissue’s oxygen supply [21].

### Studied population

This study was designed as an open-label, single-center, prospective, observational study to access the safety, feasibility, accuracy, and precision of the HSI-MIS. It is a stage 2a study according to the IDEAL framework [44]. Included in this study were 19 patients aged 18 years or older undergoing upper (*n* = 9) or lower (*n* = 10) gastrointestinal resections at the University Hospital of Leipzig between 10/09/2021 and 11/02/2022. Exclusion criteria were patients unable to consent and pregnancy.

The institutional review board of the University of Leipzig approved this study in an amendment to ethics agreement 026/18-ek, approved initially on 31/01/2018 and registered at clinicaltrials.gov (NCT04230603) on 01/13/2020. Written informed consent was obtained from all patients.

We recorded clinical and paraclinical data, such as gender and age, underlying disease, and comorbidities, specific intraoperative parameters, such as oxygen saturation (SpO_2_), hemoglobin before and after the HSI measurements were taken, catecholamine application, and postoperative complications according to the Clavien-Dindo-Classification (CDC) [20].

### Technology

**Fig. 2:**
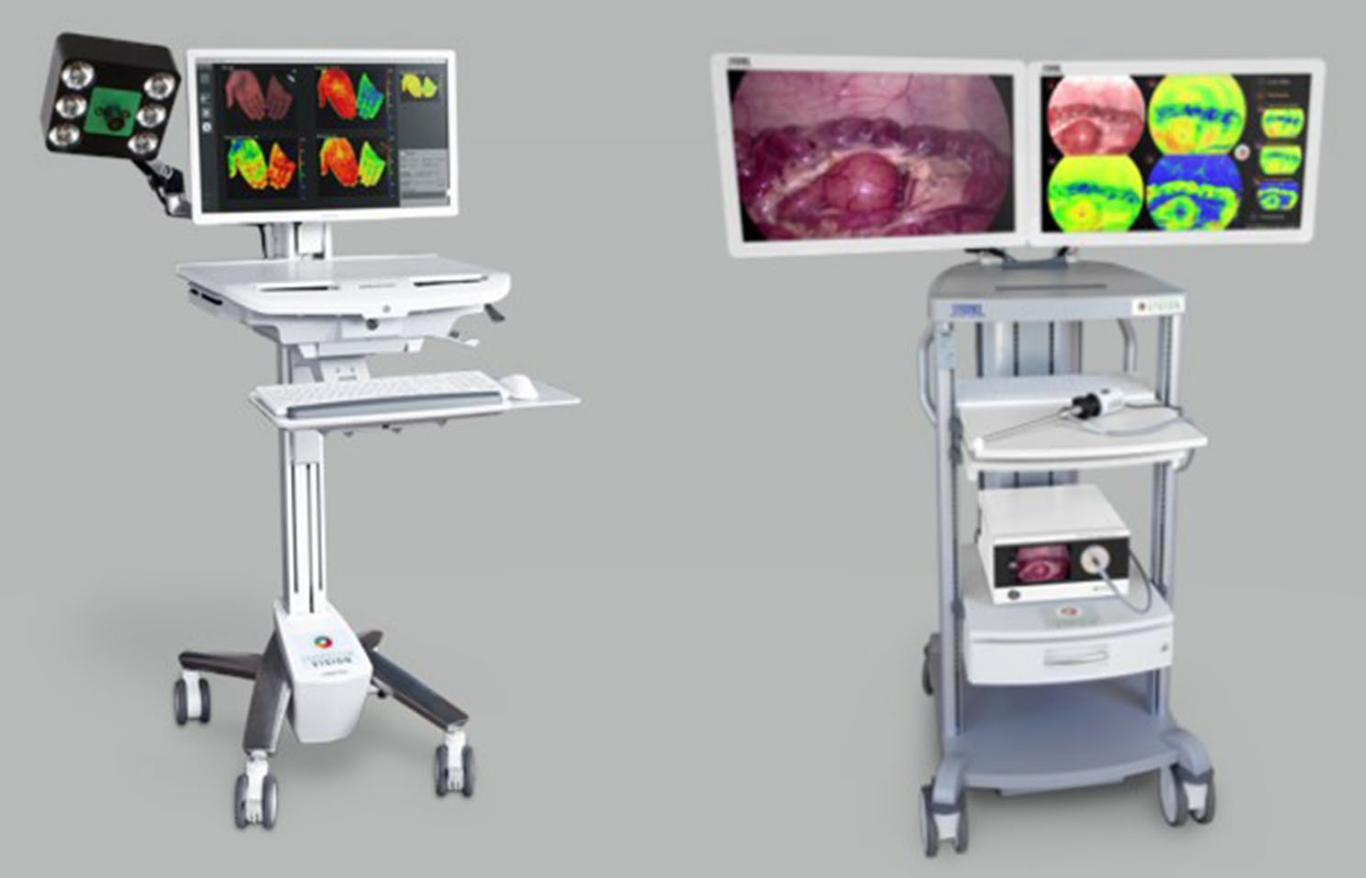
TIVITA® Tissue (left) and TIVITA® Mini Endoscopy Edition (right). Images provided by Diaspective Vision GmbH, Am Salzhaff-Pepelow, Germany

In this study, we used these HSI-system equipments (Fig. 2): the TIVITA® Tissue camera for open surgery (HSI-Open) and TIVITA® Mini Endoscopy Edition for minimally invasive surgery (HSI-MIS) (both systems from Diaspective Vision GmbH, Am Salzhaff-Pepelow, Germany). The cameras use a push-broom scanning system. A push-broom system, also known as line scanning system, collects spectral information of one line simultaneously, and moves transversally the slit to complete the hyperspectral data cube acquisition [45]. It collects reflected light at wavelengths between 500 and 1000 nm. Within this range, 100 spectral channels are recorded, giving a spectral resolution of 5 nm. The set-up of a prototype of the HSI-MIS underwent technical evaluation by Köhler et al. [41].

There are five main differences between the two systems (Table 1).

**Table 1:** Differences between the HSI-MIS and the HSI-Open

	HSI-Open	HSI-MIS
Camera size	13 × 9 × 9 cm^3^	Regular laparoscopic camera case (10 × 5 × 5 cm^3^)
Light source	6 halogen spots	Light-emitting diodes (LED)
Acquisition times	Appr. 10 s	7 s
Field of view (HSI)	8 × 6.5 cm^2^ at 50 cm distance	3.4 × 2.5 cm^2^ at 5 cm distance
Color Video	No	Yes

The camera size was reduced from 13 × 9 × 9 cm^3^ for the camera integrated into the HSI-Open into the size of a regular laparoscopic camera (10 × 5 × 5 cm^3^) in the HSI-MIS. While the HSI-Open uses six halogen spots, the HSI-MIS uses broadband Light-Emitting Diodes (LED) embedded in the computing unit positioned on a mobile cart with light transmitted via a light conductive cable. The HSI-Open’s longer acquisition time (10 s) has been shortened to 7 s in the HSI-MIS. All laparoscopic measurements were taken with a 0° to 10 mm rigid laparoscope (HOPKINS® 8711 AGA, KARL STORZ SE & Co. KG, Tuttlingen, Germany) suitable for white light and near-infrared fluorescence imaging. As expected, the field of view in the laparoscopic camera is smaller than with the HSI-Open. Furthermore, the laparoscopic camera provides a color video as needed when performing minimally invasive surgery (MIS). The video runs at 55 fps and provides full-HD images with 1920 × 1080 pixels.

### Intraoperative HSI

**Fig. 3:**
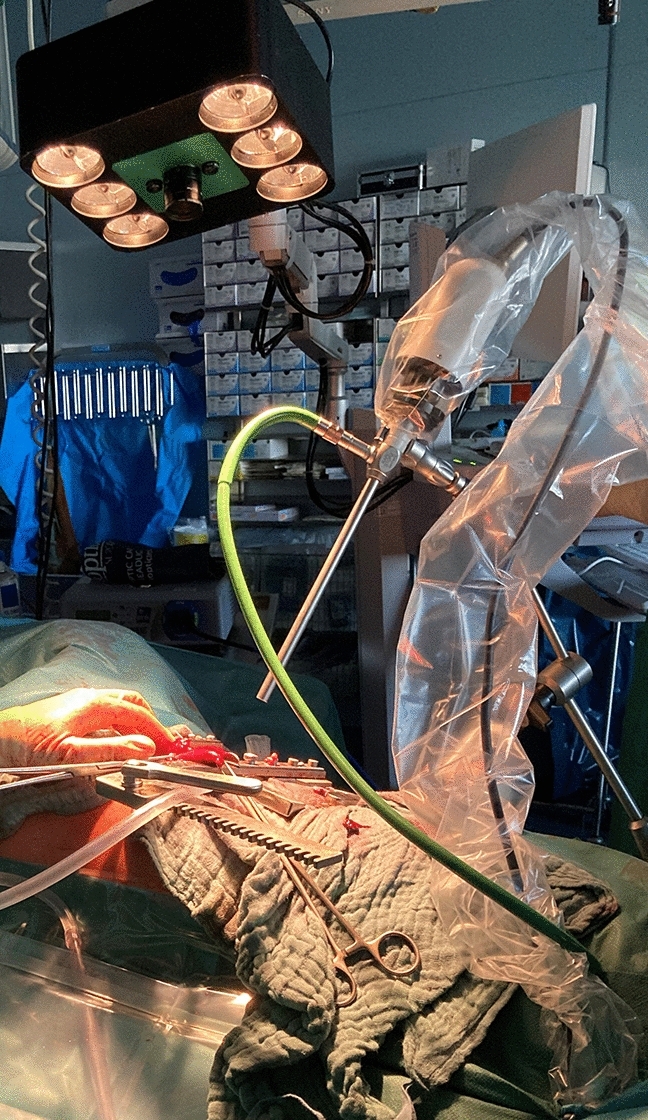
The set-up shortly before taking the measurements. During data acquisition the room was darkened

After having extracted the specimen and before the anastomosis, HSI was performed at the Region of Interest (ROI), which was directly proximal to the resection line (anastomosis location). All measurements were taken extraabdominally/extrathoracically. The ROI was subsequentially imaged with both camera systems, and we captured at least one image per camera. The laparoscope was placed in a holding device to ensure a fixed camera position, as shown in Fig. 3. The distance between the laparoscope’s tip and the ROI was kept constant at 5 cm. The room was shaded by turning off all room lights and closing the window blinds to eliminate spectral artifacts from external illumination, according to our standard protocol for intraoperative HSI measurements. Because of the unavailability of long (30 cm), and angled laparoscopes at the beginning of our study, we did not use the HSI-MIS during the whole procedure. Another reason for this was to reduce artifacts from, for example, fogging or stains on the camera lens. A similar set-up for ex vivo measurements was described by Pfahl et al. [46].

### Data analysis

**Fig. 4:**
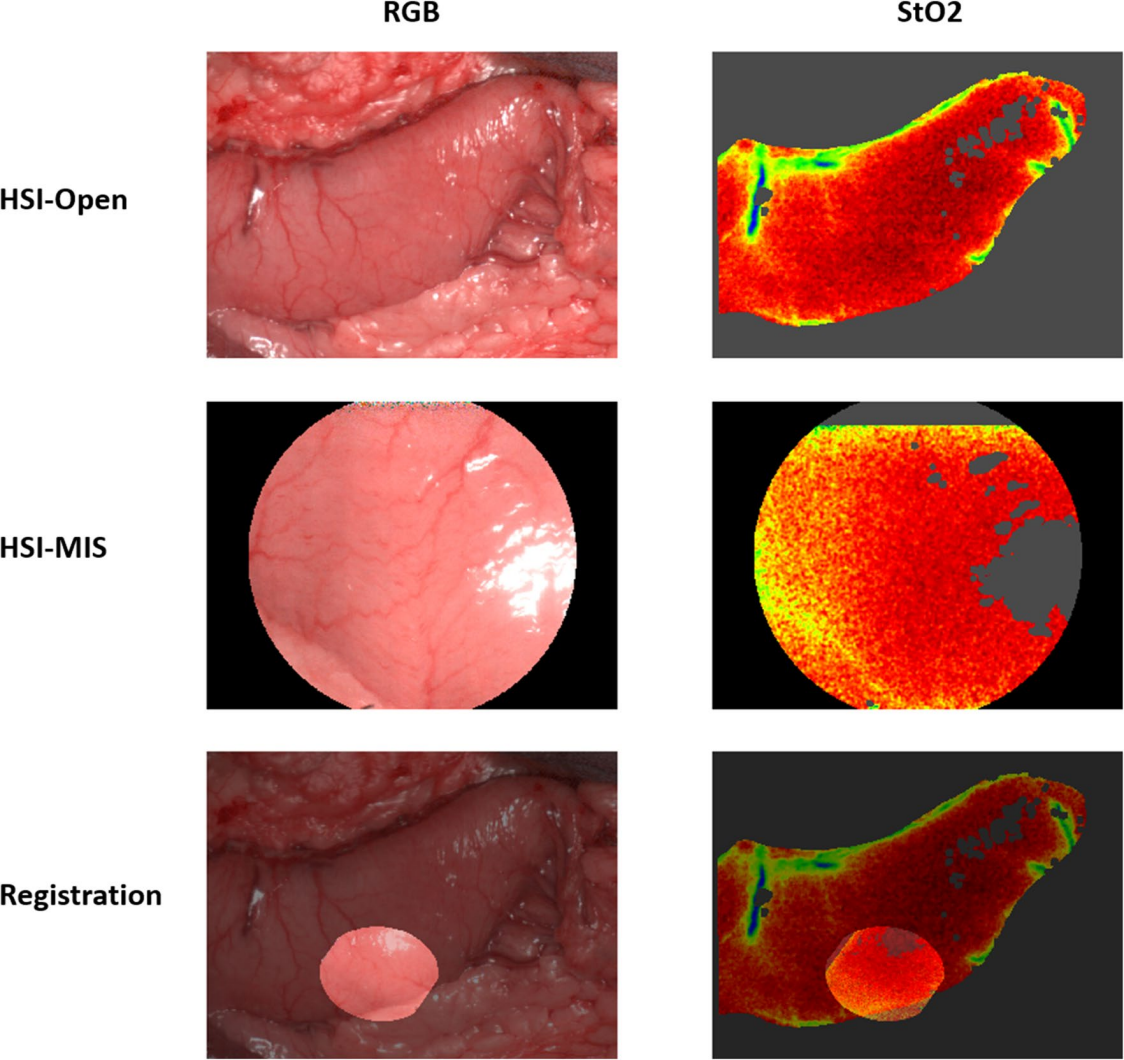
Example of a completed registration. 25 points were annotated manually on both the Red–Green–Blue-image (RGB) from the HSI-MIS and the HSI-Open. An overlay was created, allowing a pixel-wise comparison of spectra

The ROI was selected by creating a mask that excluded all image sections not showing the intestine near the resection line using the software ImageJ [47]. The images acquired with both camera systems were aligned by manually annotating 25 corresponding points and transforming the subsequent perspective (Fig. 4). Homography was obtained using OpenCV and RANSAC [48, 49] to calculate the 3 × 3 transformation matrix between two planes by minimizing the back-projection error. The spectra were compared per pixel by calculating the Rooted Mean Squared Error (RMSE) for the visible and near-infrared ranges. Furthermore, we calculated the Mean Absolute Error (MAE) for each tissue parameter from the absolute error between the corresponding pixels in the records. Both RMSE and MAE were averaged over all records.

The parameter calculation method used for the HSI-MIS had been developed for the HSI-Open initially and translated to the new system. To determine whether the error could be lowered by optimizing the parameter calculation method, we recalculated the parameters using partial least squares (PLS) regression [50]. PLS regression is widely used in chemometrics for multivariate spectroscopic data analysis. The spectra acquired with the HSI-MIS were used to build the matrix of predictors, while the matrix of responses included the tissue parameters obtained from the HSI-Open. K-fold cross-validation with *k* = 10 was used to determine the pre-processing and model parameters resulting in the highest determination coefficient *R*^2^ and lowest RMSE. Nineteen PLS models were obtained during a Leave-one-patient-out cross-validation (LOOCV) and used to predict the tissue parameters from the hypercubes not used to build our model.

**Fig. 5:**
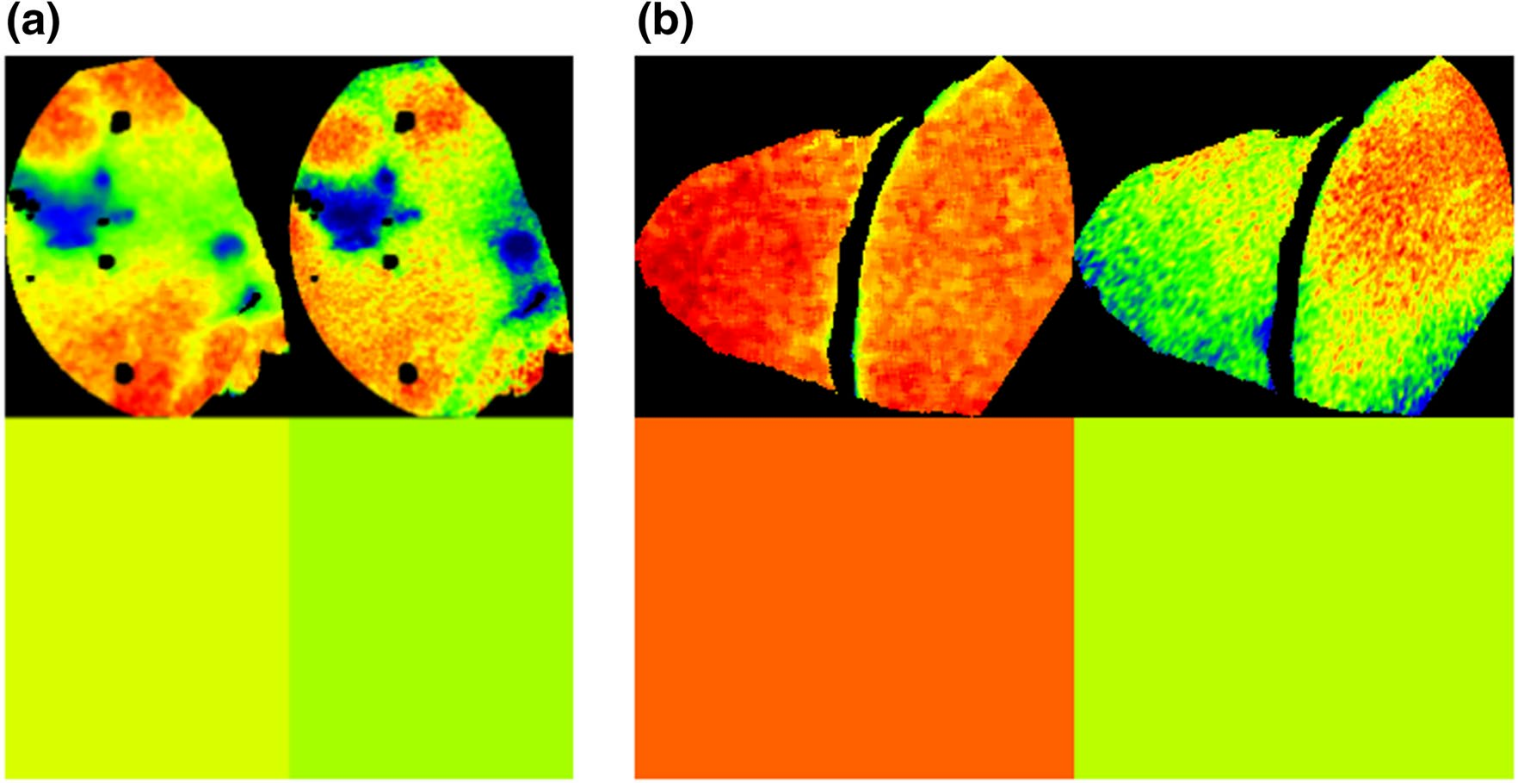
StO_2_ in matching areas (ROI, upper image) and color tiles visualizing the mean StO_2_ (lower, square image) for the HSI-Open and the HSI-MIS. In the case of **a**, the images were accepted as showing the same visual expression, in the case of **b** the image was rejected for making different visual impressions (Color figure online)

Surgeons often prefer to rely intraoperatively on their visual impression rather than on numbers on a screen. To create a more realistic setting reflecting intraoperative procedures, two senior abdominal surgeons with over 5 years of experience with intraoperative HSI (YM and BJW) conducted an additional analysis. The ROIs from both systems were visually compared, as were color tiles representing the mean value for a parameter in an image. Clinically relevant color changes were those from red to yellow, green, or blue, from orange to just yellow, green, or blue, and from yellow to green or blue (Fig. 5). Red and orange represented well perfused tissue, whereas yellow, green, and blue indicated poorer perfusion. The images were rejected if the ROIs or the color tiles changed relevantly. If not, they counted as being accepted. The same analysis was carried out using the recalculated parameters from the PLS method. Before the examination, the images were randomized, and we conducted a single-blinded analysis to ensure that the surgeons had not preferred either the original or the PLS calculation method. The images were randomly placed in folders and the original and PLS images were given the same name. The average number of images rejected and accepted per surgeon was calculated, and the average MAE of the rejected and accepted images was compared. Cohen’s *κ* coefficient was calculated to measure the level of interobserver agreement [51]

## Results

**Fig. 6:**
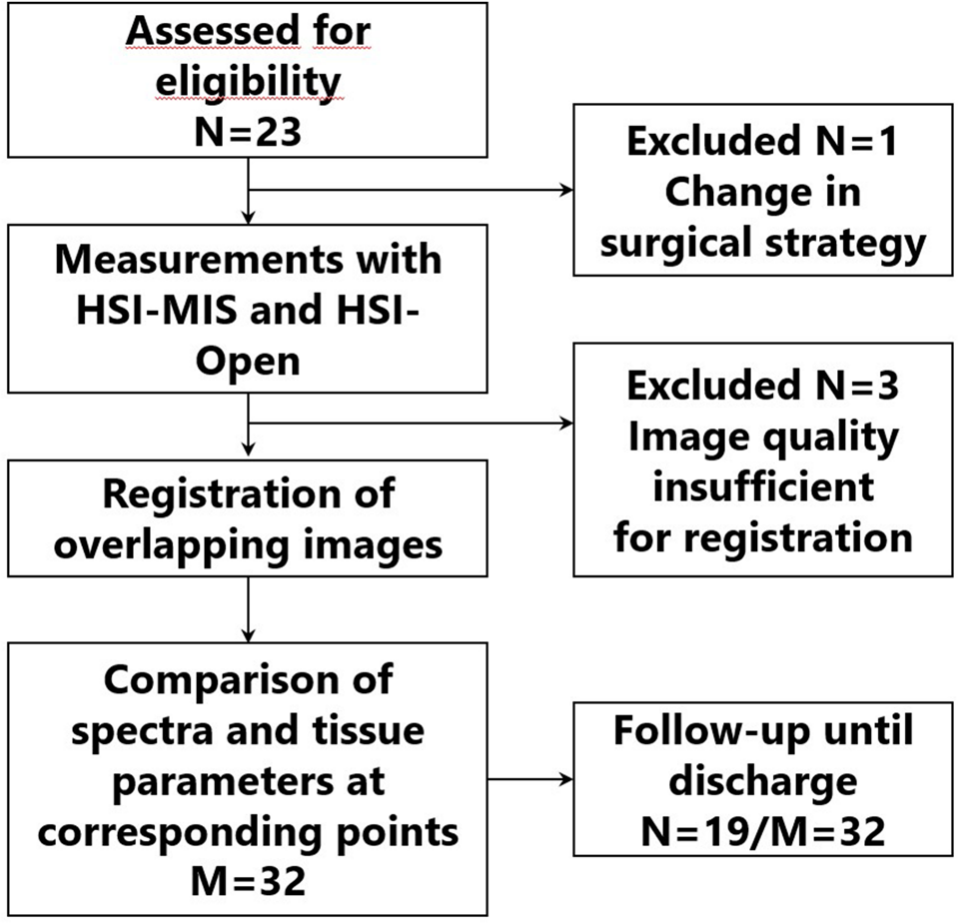
The study’s flow chart visualizes patient selection and trial set-up, with *N* = number of patients and *M* = number of images registered successfully

The study flow chart in Fig. 6 visualizes our set-up and patient selection. Twenty-three patients were eligible for the study. Four were excluded because the surgical strategy had to be changed before the measurements were performed (*N* = 1), and the HSI-MIS image quality was insufficient or out of focus (*N* = 3). Included patients underwent esophagectomy (*N* = 9), proctocolectomy (*N* = 1), hemicolectomy (*N* = 7) and low anterior rectal resection with total mesorectal excision (TME) (*N* = 2). Seventeen patients underwent oncological resections, one underwent hemicolectomy for abscess removal and another one proctocolectomy for ulcerative colitis treatment. The average age of patients was 63.1 (± 14.4) years, with an average BMI of 28.2 (± 6.1) kg/m^2^. Selected patient characteristics are listed in Table 2.

**Table 2:** Selected patient characteristics

Patient Characteristics (Total *N* = 19)	*N*
Female	6
Male	13
BMI > 30 kg/m^2^	6
Arteriosclerosis	5
Arterial Hypertension	16
Neoadjuvant Chemotherapy	13
ASA I	1
ASA II	9
ASA III	9

HSI was safe and feasible in all 19 patients. The intraoperative procedures and surgical workflow went smoothly in all patients, as the HSI measurements (by conventional and miniature systems) took only few seconds to acquire visual information and process data. Minor incidents included connectivity problems requiring the restart of the HSI-MIS, and surgery prolonged by about one minute per restart. As the images had revealed many movement artifacts during the preliminary measurements, we later had to use a laparoscopic holding device.

Thirty-two images (M) were collected and registered to compare spectra and tissue parameters at corresponding points. An average of 20,146 (± 7,996) spectra were obtained per patient.

**Fig. 7:**
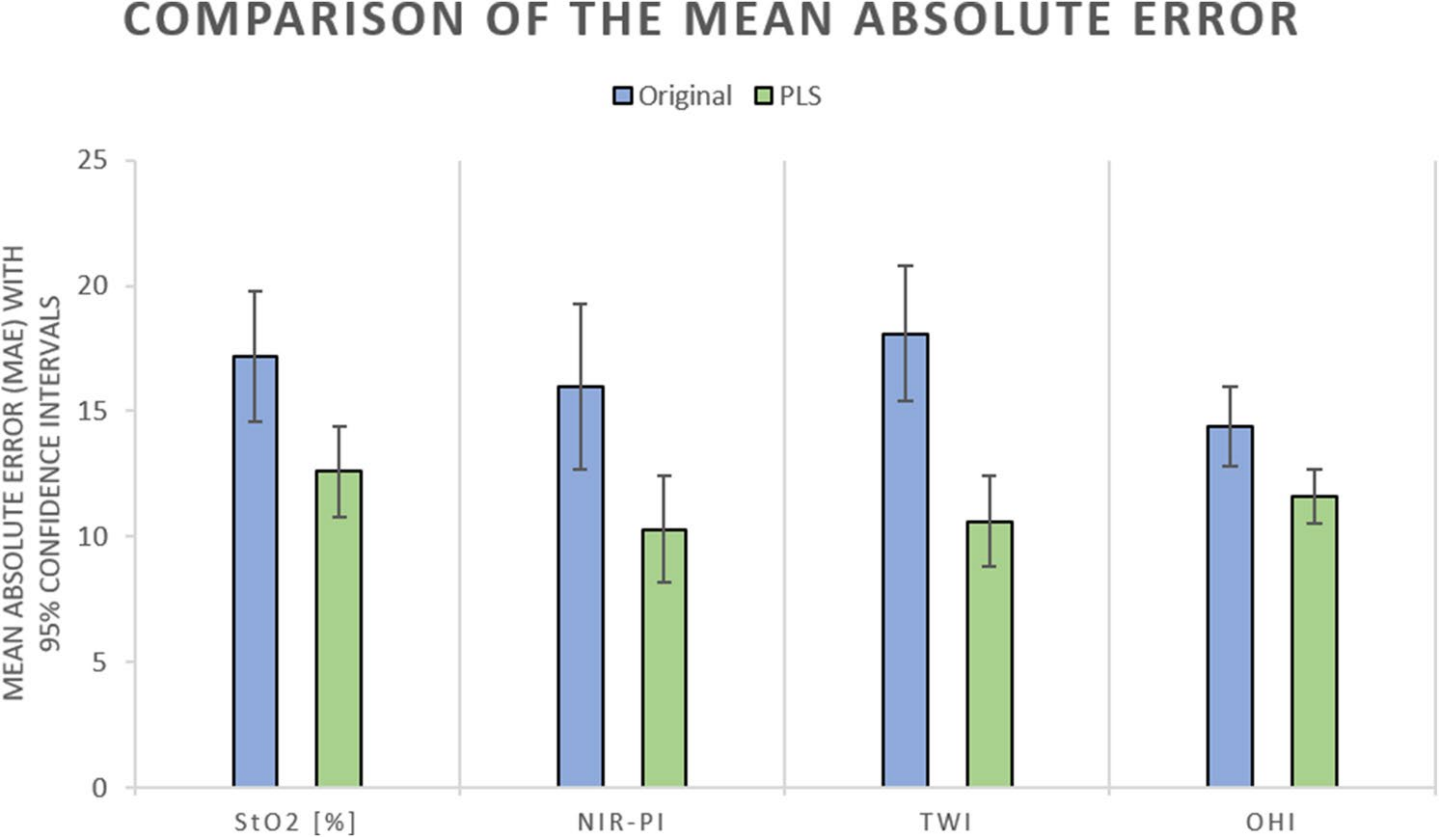
Mean absolute error (MAE) with 95% confidence intervals for the original (= manufacturer’s, orig.) calculation and the calculation using partial least squares (PLS). The confidence intervals were StO_2 orig._ (14.6%, 19.9%), StO_2 PLS_ (10.8%, 14,4%), NIR-PI_orig._ (12.7, 19.3), NIR-PI_PLS_ (8.2, 12.3), TWI_orig._ (15.4, 20.9), TWI_PLS_ (8.8, 12.3), OHI_orig._ (12.8, 16.0), OHI_PLS_ (10.5, 12.6)

MAE values (Fig. 7) between the HSI parameters provided by the HSI-Open and HSI-MIS system and using the manufacturer’s (original) and the PLS method were StO_2 orig._ 17.2% (± 7.7%)_,_ StO_2 PLS_ 12.6% (± 5.2%), NIR-PI_orig._ 16.0 (± 9.5), NIR-PI_PLS_ 10.3 (± 6.0), TWI_orig._ 18.1 (± 7.9), TWI_PLS_ 10.6 (± 5.1), OHI_orig._ 14.4 (± 4.5), and OHI_PLS_ 11.6 (± 3.0).

These were remarkably reduced when using the PLS method, with improvements ranging from 19.2 to 42.3% when compared to the original calculation (Fig. 7). The TWI revealed the largest improvement for and the OHI the smallest.

The mean RMSE value between spectra was 0.14 (± 0.06), with a mean RMSE of 0.13 (± 0.06) in the visual range (500–750 nm) and a mean RMSE of 0.16 (± 0.06) in the near-infrared (750–100 nm) range.

**Fig. 8:**
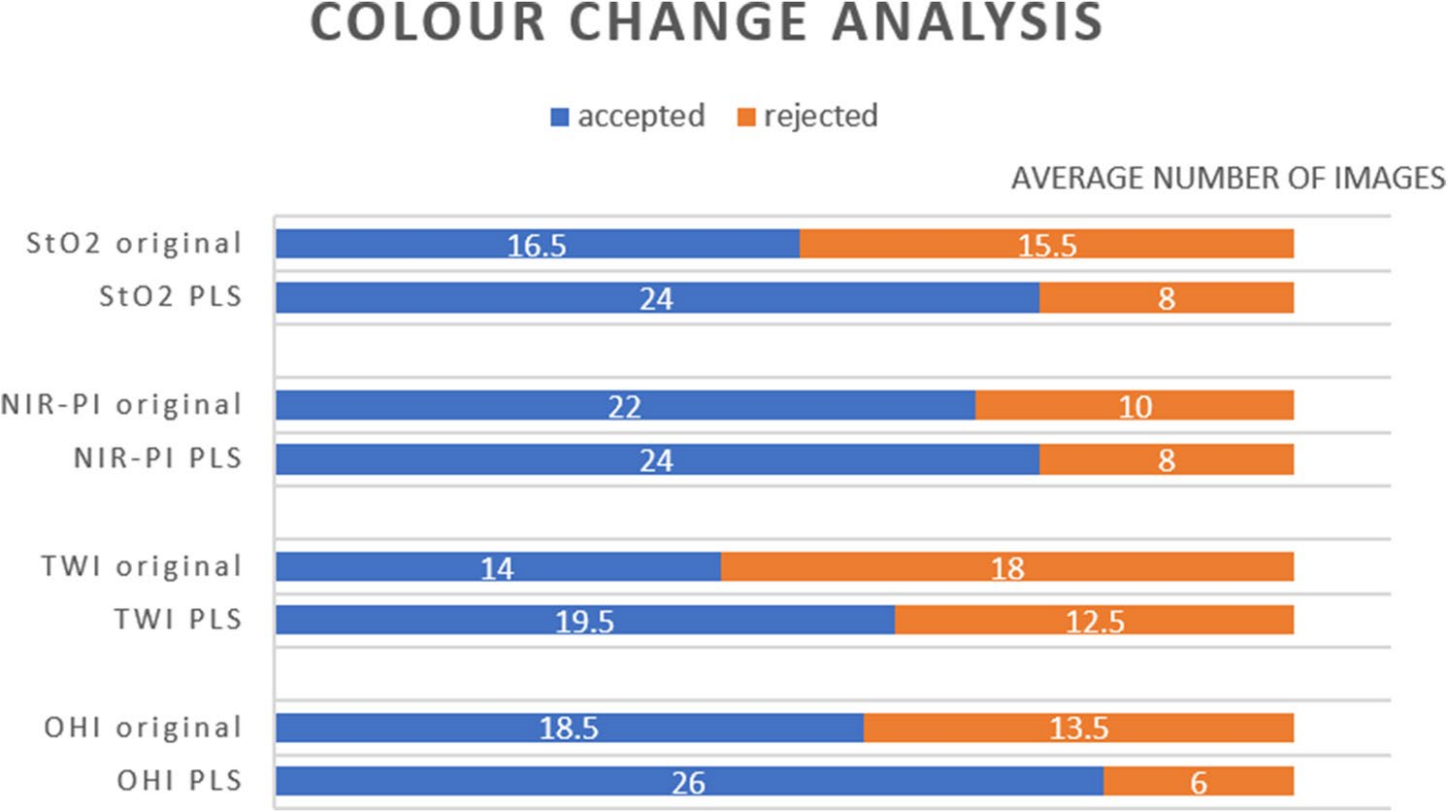
Color change (visual) comparison of parameter images at the ROI and color tiles visualizing the mean value of a parameter at the ROI via the original (= manufacturer’s) calculation vs. the optimized calculation using the PLS method for Oxygen Saturation (StO_2_), Near-Infrared Perfusion Index (NIR-PI), Tissue Water Index (TWI) and Organ Hemoglobin Index (OHI)

Concerning our color-change analysis results, the PLS method proved to raise the average number of images accepted for all parameters (Fig. 8). Improvement was greatest for the StO_2_ and OHI, and smallest for the NIR-PI. We noted a high level of interobserver variability (Table 3) with Cohen’s coefficient *κ* = 0.25. 87.5% of those images rejected by surgeon one were also dismissed by surgeon two. Surgeon two rejected many other images: while surgeon one rejected 48 out of 256 images, surgeon two rejected 135 out of 256 images. However, both surgeons accepted more images when they had been calculated via the PLS method. For the original method, the accepted images of all parameters had an average MAE of 11.0 (± 4.6) units, whereas the average MAE of all rejected images was 19.5 (± 7.3) units. For the PLS method, it was 9.3 (± 3.2) versus 14.0 (± 5.9) units.

**Table 3:** Number of images accepted/rejected per surgeon. Both surgeons accepted the same 115 and rejected the same 42 images

		Surgeon 2		
		Accepted	Rejected	Total
Surgeon 1	Accepted	115	93	208
	Rejected	6	42	48
	Total	121	135	256

Surgeon 1 accepted additional 93 images, that Surgeon 2 rejected, whereas Surgeon 2 accepted 6 images, that Surgeon 1 rejected

## Discussion

Our study aims to enable the first translation of HSI for MIS within an in vivo setting. Its use proved to be safe and feasible in all patients. The original calculation’s MAE amounted to between almost 15 units or slightly above that. The error was greatly reduced to around 10 units using a different calculation mode (the PLS method). This finding was supported by the (blinded) visual analysis of the HSI parameter images by two experienced surgeons. While only 15.5 out of 32 StO_2_ images were accepted on average when computed using the original method, 24 were accepted after optimization with the PLS model. The OHI, TWI, and NIR-PI revealed similar improvements. Surgeon two rejected almost all those images rejected by surgeon one and many additional images. However, both surgeons rejected fewer images that had been calculated via the PLS method.

This study is the first to evaluate in vivo an HSI camera designed for routine use in MIS. It is an essential step toward the clinical implementation of laparoscopic hyperspectral imaging. We were able to compare a vast number of spectra per patient. Our findings create the framework for future, more extensive studies using HSI-MIS and approximating the expected error. Our investigation enabled the gathering of information about handling this system, and revealed minor technical limitations, such as connectivity issues, which were reported and fixed later by the company. We also managed to define means of improvement, namely by optimizing the calculation method.

Preliminary testing prior to this study revealed parameter images with many movement artifacts when the HSI-MIS is manually held. These affected only the spatial information, while the spectral information remained correct. To obtain images suited for matching, we placed the laparoscope in a holding device. Surgeons need a similar device to obtain equally accurate images. For clinical surgery, where no matching is required, a surgeon can obtain well interpretable hand-held images, if the laparoscope is held still. We recommend that future cameras are outfitted with an image-stabilization function.

Limitations of this pilot study are the limited number of patients included and the setting. Although performed on in vivo tissues, our study did not precisely mimic HSI’s intraoperative laparoscopic application, since the measurements were taken extracorporeally (extraabdominally/extrathoracically) during resections. Currently, both HSI and ICG-FA are established methods for measuring the perfusion of abdominal organs intraoperatively. Although ICG-FA is used more regularly in many clinics, we did not use it as a reference imaging method, since the two modalities measure different parameters and use different technology. Quantitative ICG-FA analyses the inflow-curve of an exogenous dye, whereas HSI measures physiological tissue parameters, such as StO_2_, TWI, OHI, and NIR-PI. Therefore, the comparison to a camera that uses the same type of technology and the same parameters seemed better suited for this first in vivo study. In order to compare it to the established HSI-system and enable a pixel-wise comparison of spectra, the same region had to be imaged without moving the tissue between measurements. Only tissue retrieved extracorporeally was measured, as we could not obtain intracorporal images with the HSI-Open. Because only a short laparoscope (20 cm) was available when the first images were acquired and to reduce artifacts, we were not able to perform the whole procedure using the HSI-MIS. Although HSI has been assessed in different surgical disciplines, its suitability for laparoscopic surgery remains limited, and no system has been adopted for routine clinical use. Prototypes of the now CE-certified HSI-MIS were recently evaluated ex vivo [41, 46], revealing an MAE of about 10 units, and higher for each parameter. Our findings for the original calculation of the physiological parameters were slightly higher than the MAE reported by Pfahl et al. [46], while the MAE matched previous results when applying the PLS method. Therefore, part of the error is inherent to the system hardware, and part is due to how the parameters are calculated. Differences in build-up comprise lenses**,** sensors, and light sources. The error resulting from calculating the parameters could be reduced, leading to a better correlation between the two systems. The influence of additional factors, such as distance between the ROI and laparoscope, was not investigated in this study. This must be done in future studies. Concerning our color-change analysis, notice that it was a subjective one carried out by two surgeons only with only a fair level of agreement. The scale was chosen by the surgeons based on their clinical expertise, thereby not externally validated. We blinded the surgeons to increase internal validity. We decided on this to simulate a setting more closely resembling actual, live surgery; our findings should be considered as a qualitative add-on analysis to objective analyses determining the MAE and RMSE.

This study’s results lay the foundation for future in vivo studies using laparoscopic HSI under real operation conditions, showing that it can be safely and efficiently implemented in MIS. Larger, multi-centered studies are needed to verify the findings of this first in vivo analysis. Another goal is to perform a whole procedure with the HSI-MIS. Future studies could also comprise perfusion measurements of in situ anastomoses and comparisons between HSI-MIS and other established methods for assessing perfusion, such as quantitative ICG-FA. Other possible applications include detecting at-risk structures and differentiating cancerous from non-cancerous tissue.

Intraoperative HSI for minimally invasive procedures is a new and promising technology holding great potential for future applications in surgery.

## Abbreviations

AL: Anastomotic leakage
ASA: American society of anaesthesiologist physical status classification
BMI: Body Mass Index
CDC: Clavien-Dindo-Classification
HSI: HyperSpectral imaging
HSI-MIS: HyperSpectral imaging system for minimally invasive surgery
HSI-Open: HyperSpectral imaging system for open surgery
CG-FA: IndoCyanine green-fluorescence angiography
LED: Light-emitting diodes
LOOCV: Leave-one-patient-out cross-validation
MAE: Mean absolute error
MIS: Minimally invasive surgery
NIR-PI: Near-Infrared Perfusion Index
OHI: Organ Hemoglobin Index
PLS: Partial least squares
RGB: Red–Green–Blue
RMSE: Root mean squared error
ROI: Region of interest
SpO_2_: Oxygen saturation
StO_2_: Tissue oxygen saturation
TME: Total mesorectal excision
TWI: Tissue Water Index
